# Effective components of *Coptidis Rhizoma* and *Cinnamomi Cortex* in the treatment of renal cell carcinoma and their mechanism of action

**DOI:** 10.1097/MD.0000000000043786

**Published:** 2025-11-21

**Authors:** Dongmei Duan, Jin Wang, Mingjun Chen, Liyuan Tan, Lingling Song

**Affiliations:** a National Clinical Research Center for Geriatric Diseases, The Second Medical Center, Chinese PLA General Hospital, Beijing, China; b State Key Laboratory of NBC Protection for Civilian, Beijing, China; c Shanghai Livzon Pharmaceutical Co., Ltd, Shanghai, China; d Beijing Tongrentang Technology Development Co., Ltd, Beijing, China.

**Keywords:** *Coptidis Rhizoma* and *Cinnamomi Cortex*, molecular docking, molecular mechanisms, network pharmacology, renal cell carcinoma

## Abstract

This study aimed to investigate the mechanism of the *Coptidis Rhizoma* and *Cinnamomi Cortex* (HL-RG) drug pair in the intervention of renal cell carcinoma (RCC) using network pharmacology, molecular docking, and cell experiments. Network pharmacology analysis predicted 42 active components in HL-RG and identified 227 potential targets. Among these, 50 targets were specifically associated with RCC, and 18 were identified as hub genes. Three components (quercetin, oleic acid, tetrandrine) were highlighted as particularly effective. The results of the gene ontology (GO) annotation showed that HL-RG may treat RCC by regulating biological processes such as inflammation, immune response, cell cycle process, and lipid metabolism. Kyoto Gene and Genome Encyclopedia (KEGG) enrichment revealed that the key targets of HL-RG in treating RCC were enriched in the proteoglycans in cancer, and the HIF-1 signaling pathway. Molecular docking results demonstrated that 87% of the interactions exhibited binding energies stronger than −5.0 kcal/mol, indicating favorable binding affinity between the core active components and key targets. Experimental validation using quantitative real-time polymerase chain reaction and Western blot in 786-O cells demonstrated that tetrandrine (TET) and quercetin (QUE) downregulated the mRNA and protein levels of G1/S-specific cyclin-D1 (CCND1) and Transforming growth factor beta-1 (TGFB1), and upregulated the mRNA levels of Catalase (CAT). Additionally, QUE downregulated the mRNA levels of Receptor tyrosine-protein kinase erbB-2 (ERBB2) and upregulated the mRNA levels of Pro-epidermal growth factor (EGF). Furthermore, oleic acid (OA), TET, and QUE downregulated the protein levels of Matrix metalloproteinase-9 (MMP9). In conclusion, the therapeutic effect of the HL-RG combination against RCC is primarily mediated by its bioactive components, QUE, OA, and TET. These components regulate the HIF-1 signaling pathway, activating genes involved in the cellular response to hypoxia and modulating the expression of proteins that control glucose metabolism, cell proliferation, and angiogenesis.

## 1. Introduction

Renal cell carcinoma (RCC), originating from renal tubular epithelial cells, is one of the common malignant tumors of the urinary system, characterized by an insidious onset and the absence of clinical manifestations in its early stages.^[1]^ Globally, it is estimated that there are approximately 431,000 new cases of renal cancer and 179,000 deaths annually.^[2]^ Epidemiological data from China indicate that renal cancer has the second highest incidence among urinary system malignancies, with approximately 66,800 new cases and 23,400 deaths each year, and its incidence continues to rise. Among genitourinary tumors, RCC ranks third in incidence but first in mortality. Surgery remains the primary treatment for early-stage and locally advanced RCC.^[3]^ Patients with advanced disease are primarily managed with tyrosine kinase inhibitors (TKIs) and mammalian target of rapamycin (mTOR) inhibitors. However, most patients eventually develop drug resistance, and these treatments are often associated with low efficiency, high toxicity, and significant side effects. Consequently, early diagnosis and improving therapeutic outcomes for RCC patients present major clinical challenges. Therefore, the development of novel biomarkers for early cancer detection and the investigation of their potential mechanisms in cancer are crucial.^[4]^

Traditional Chinese medicine (TCM) can play a significant role in preventing postoperative recurrence of RCC, delaying treatment resistance, and alleviating side effects caused by Western medicine. This approach may prolong survival time, improve patients’ quality of life, and has shown positive clinical outcomes. Professor Zhu Shijie believes that the treatment of RCC should be based on Wenyang Sanjie (Wenyang refers to the treatment of expelling cold pathogens by warming and tonifying yang qi, while Sanjie refers to the treatment of eliminating lumps by drugs that dispel blood stasis),^[5]^ Professor Wang Mianzhi believes that the treatment of kidney diseases should strengthen the body resistance and eliminate pathogenic factors, regulate the spleen and stomach, and treat it steadily and slowly.^[6,7]^ Professor Wang used *Epimedii Folium* and *Curculiginis Rhizoma*, *Coptidis Rhizoma* and *Cinnamomi Cortex* (HL-RG) and so on very skillfully and effectively, and prescription composed of 2 recipes of herbs.

Studies have shown that the Wenyang Sanjie recipe, which includes *Epimedii Folium* and *Curculiginis Rhizoma* to tonify kidney-yang and dispel cold-dampness, is effective in treating renal cancer. Modern pharmacological studies have also confirmed that the combination of *Epimedii Folium* and *Curculiginis Rhizoma* can inhibit the proliferation, metastasis and invasion of tumor cells, and enhance the cellular immune function to achieve the anti-cancer effect of “eliminating pathogenic factors and strengthening the body resistance.”^[8,9]^ The mechanism of its treatment of renal cancer has been reported. HL-RG was first published in *Hanyitong* (A book written by Han Mao in 1522), *Coptidis Rhizoma* (HL) is the Rhizoma of the plant *Coptis chinensis* and *Cinnamomi Cortex* (RG) is the dried inner bark of the plant *Cinnamomum cassia*, and later renamed as Jiaotai Pill. Both of them are used to treat renal diseases such as palpitation, insomnia, dreaminess, and other cardiac and renal disorders caused by disharmony between the heart and kidney. At present, the research on it mainly focuses on diabetic nephropathy. Modern pharmacological research also shows that both HL-RG has anti-tumor and anti-inflammatory properties, but the work on its mechanism of action on renal cancer has not been reported.

**Figure 1.**
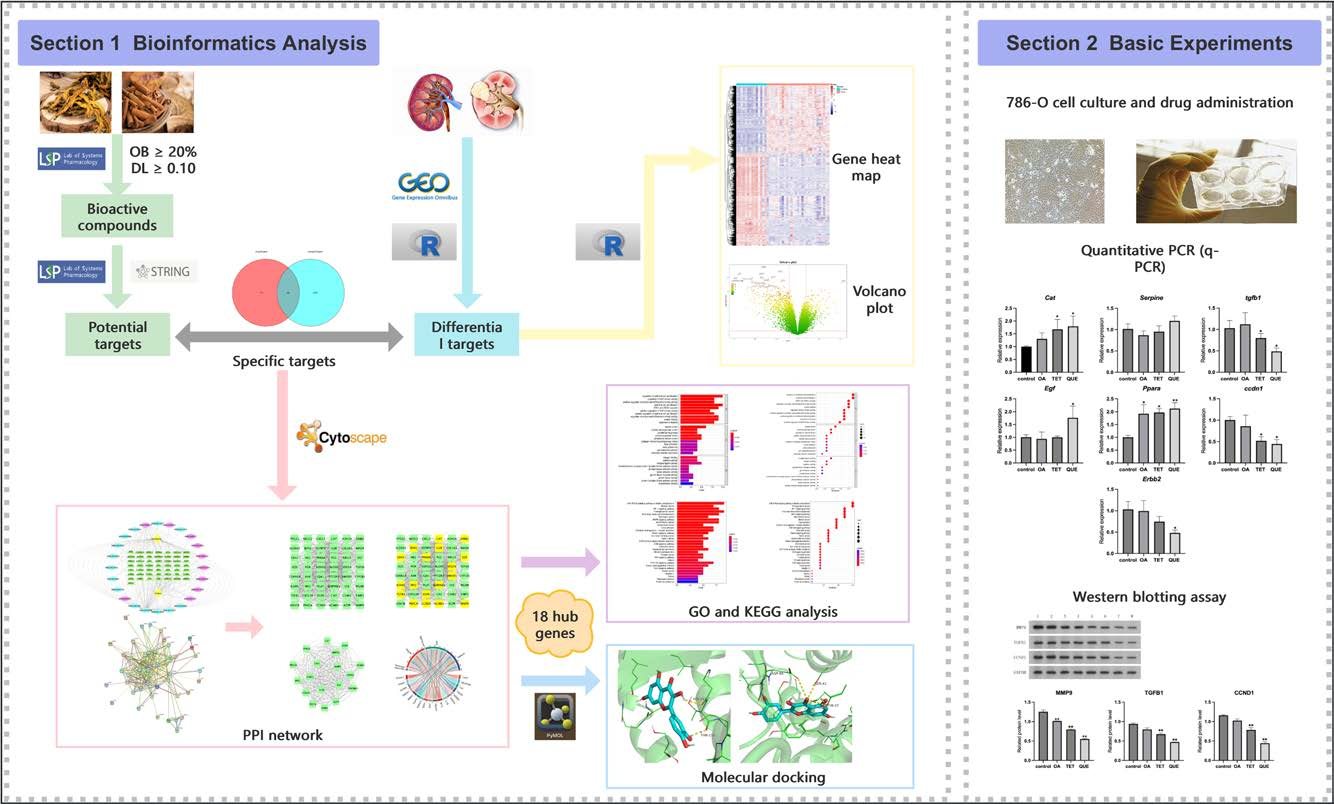
Research workflow. HL-RG in the treatment of RCC and its mechanism are shown in the figure using bioinformatics analysis and experiments. HL-RG = Coptidis Rhizoma and Cinnamomi Cortex, RCC = Renal cell carcinoma.

In this study, we selected the HL-RG drug pair and employed network pharmacology and molecular docking technology to analyze its mechanism in treating RCC at the molecular level. Our objectives were to identify potential active components, explore RCC biomarkers and its carcinogenic mechanisms, and provide a theoretical basis for TCM-based clinical treatment of RCC. Finally, 3 key bioactive components were screened: oleic acid (OA), tetrandrine (TET), and quercetin (QUE). QUE, a naturally occurring flavonoid, exhibits antioxidant, anti-inflammatory, and anti-tumor activities. TET, an alkaloid from the *Menispermaceae* family, possesses anti-tumor and immunomodulatory effects. OA, a monounsaturated fatty acid, improves lipid metabolism and has demonstrated in vitro anti-cancer potential. Some studies have also investigated the mechanism by which OA inhibits cancer cells at the cellular level in vitro, suggesting the potential for OA in cancer treatment. We verified the effects of QUE, TET, and OA on RCC through in vitro cell experiments. Quantitative real-time polymerase chain reaction (qRT-PCR) and Western blot results confirmed the mRNA and protein expression levels of pathway genes, consistent with bioinformatics analysis. Additionally, we analyzed differential genes between RCC patients and healthy controls to discuss potential biomarkers. A graphical abstract of the HL-RG mechanism in RCC treatment is provided (Fig. 1).

## 2. Materials and methods

### 2.1. Acquisition, screening and network construction of HL-RG bioactive compounds and their related human targets

Using “huanglian” (*Coptidis Rhizoma*) and “rougui” (*Cinnamomi Cortex*) as keywords, we searched the TCM Systems Pharmacology (TCMSP) database^[10]^ (https://www.tcmsp-e.com/) to retrieve all bioactive components. Initial screening criteria were oral bioavailability (OB) ≥ 20% and drug-likeness ≥ 0.10. Through literature review, additional components of *Cinnamomi Cortex* – including styrone, cinnamaldehyde, cis-cinnamaldehyde, and cinnamic acid – were included. Targets corresponding to these components were identified via TCMSP, and their protein names were standardized using the STRING database (https://string-db.org) with “Homo sapiens” selected as the organism. The interrelated network of TCM-bioactive components-target was constructed by Cytoscape software (version 3.7.2), and the results were visualized.

### 2.2. The specific target of RCC

We queried the Gene Expression Omnibus (https://www.ncbi.nlm.nih.gov/geo/) database using “renal cell carcinoma” as the search term.^[11,12]^ Data were filtered by selecting “Series,” “Expression profiling by array,” and “Homo sapiens.” The microarray dataset GSE68417 (submitted by Thibodeau et al^[13]^), generated on the GPL6244 platform ([HuGene-1_0-st] Affymetrix Human Gene 1.0 ST Array), was analyzed. This dataset comprised 49 samples, with 6 benign kidney conditions excluded. We compared RCC samples (Fuhrman grades 1–4, n = 29) to normal kidney samples (n = 14). Data preprocessing was performed in R Studio (version 3.7.2), and differential targets were identified using the Limma package with cutoff thresholds of |log_2_ fold change (logFC)| ≥ 1 and *P*-value (*P*) ≤ .05. The processed data are visualized by R package of Gene heat map and volcano map.

### 2.3. Network pharmacology analysis of HL-RG in RCC treatment: target identification, protein–protein interaction (PPI) network construction, and functional enrichment

#### 2.3.1. Identification of HL-RG-related RCC targets and construction of Chinese medicine-compound-target network

The RCC gene expression matrix was retrieved from Gene Expression Omnibus, and differential analysis identified specific genes. Through Venny analysis, we intersected these genes with HL-RG targets to obtain disease-drug-specific targets. Using R packages, we correlated targets with HL-RG compounds and constructed a Chinese Medicine-Compound-Target network in Cytoscape (version 3.7.2).^[14]^

#### 2.3.2. Construction of PPI network and hub gene screening

The related targets were uploaded to the STRING database (species: “Homo sapiens”). Target interactions were extracted and stored as a TSV file. Using R Studio, we calculated the “betweenness,” “closeness,” “degree,” “eigenvector,” “LAC,” and “network” scores to identify hub genes. The hub gene interaction network was visualized using Cytoscape software (version 3.7.2).^[15]^

#### 2.3.3. Functional annotation and pathway analysis gene

Gene ontology (GO) functional annotation and Kyoto Gene and Genome Encyclopedia (KEGG) pathway analysis were performed using R packages. We set the *P*-value cutoff at <.05 and selected “org.Hs.e.g..db” as the species-specific database.^[16]^ For GO analysis, the top 10 enriched terms in biological process (BP), cellular component (CC), and molecular function categories were selected based on *P*-value.^[17]^ For KEGG analysis, the top 30 pathways were visualized. Finally, we constructed a hub gene-pathway network comprising 30 key pathways and 18 hub genes using Cytoscape (version 3.7.2).^[18]^

### 2.4. Compound-target molecular docking

The 3D structures of the primary active components of HL-RG were obtained from the PubChem database (https://pubchem.ncbi.nlm.nih.gov/), and their molecular energies were minimized using ChemBioOffice software. The 3D structures of HL-RG-processed target proteins were downloaded from the RCSB Protein Data Bank (https://www.rcsb.org/).^[19]^ These structures were imported into PyMOL, AutoDockTools, and AutoDock Vina for processing, including removal of small molecular ligands and water molecules, hydrogenation, and subsequent receptor-ligand docking. The binding affinity and stability between targets and active components were evaluated based on docking scores and hydrogen bond formation. Final docking results were visualized using PyMOL software.

### 2.5. Validation of hub target gene expression

#### 2.5.1. 786-O cell culture and drug administration

Human renal carcinoma cells (786-O) utilized in this study were commercially obtained from Shanghai Zhong Qiao Xin Zhou Biotechnology Co., Ltd (Shanghai, China). As these are established, anonymized cell lines not derived specifically for this research, ethical approval was not required by the Institutional Review Board of the Chinese People’s Liberation Army (PLA) General Hospital in accordance with institutional policies on the use of commercially available cell lines. And cultured in Dulbecco modified Eagle medium (DMEM, Gibco, Grand Island) supplemented with 10% fetal bovine serum (FBS, Gibco) and 1% penicillin-streptomycin (Gibco) at 37°C with 5% CO_2_.^[20]^ For each experimental condition, 3 biological replicates were performed independently. Cells were seeded in 6-well plates (2.5 × 10^5^ cell/well, with 3 technical replicates per group), and allowed to adhere for 24 hours before treatment. After discarding the culture medium, cells were divided into: Control group (0.1% DMSO), OA group (oleic acid, 50 μM), TET group (tetrandrine, 50 μM), and QUE group (quercetin, 50 μM).

#### 2.5.2. qRT-PCR analysis

Some related genes were selected from differentially expressed genes and verified by qRT-PCR. Total RNA was isolated from 3 biological replicates (each with 3 technical replicates) using RNA FAST 200 reagent Kit (220011, FASTAGEN, Shanghai, China). Reverse transcription was carried out according to the instructions of PrimeScript^TM^ RT reagent Kit with gDNA Eraser (Takara) and SYBR Green Premix Pro Taq HS qPCR Kit (AG11701, Accurate Biotechnology, Changsha, Hunan, China) was used for qRT-PCR. The results were calculated using the 2^-ΔΔCt^ method.^[21]^ *Gapdh* (Glyceraldehyde-3-phosphate dehydrogenase) as a reference gene. The specific primer sequences used were as follows: *Tgf-β1* (Transforming growth factor beta-1): forward: 5′-GAAAGCCTGCCGGTGACTAA-3′, reverse: 5′-GCATCACCCGGAGGAGAAAT-3’; *Ccnd1* (G1/S-specific cyclin-D1): forward: 5′-GAGGCGGAGGAGAACAAACA-3′, reverse: 5′-GGAGGGCGGATTGGAAATGA-3’; *Cat* (Catalase): forward: 5′-CTGTTGCTGGAGAATCGGGT-3′, reverse: 5′-AGGACGTAGGCTCCAGAAGT-3’; *Erbb2* (Receptor tyrosine-protein kinase erbB-2): forward: 5′-GGTGTGAGAAGTGCAGCAAG-3′, reverse: 5′-GCACTGGTAACTGCCCTCAC-3’; *Ppar*α (Peroxisome proliferator-activated receptor alpha): forward: 5′-GCTGGTGTATGACAAGTGCG-3′, reverse: 5′-TGACATCCCGACAGAAAGGC-3’; *Egf* (Pro-epidermal growth factor): forward: 5′-CCCCAGGTAATGGAGCGAAG-3′, reverse: 5′-ATTTGGTGTGGTGGGTCCAG-3’; *Serpine1* (Plasminogen activator inhibitor 1): forward: 5′-TCTCAGGAAGTCCAGCCACT-3′, reverse: 5′-CCCCACTCCGTCCTTTTGAT.

#### 2.5.4. Western blotting assay

Proteins were extracted from cells with RIPA (R0020, Solarbio, Beijing, China). Protein concentrations were determined with a BCA protein detection kit (PICPI23223, Thermo, Shanghai, China). Quantified protein samples were treated with SDS-PAGE, transferred onto PVDF membranes, and sealed with skim milk (5%). PVDF membranes were subsequently incubated with the corresponding primary antibody overnight at 4°C, followed by incubation with an HRP-bound secondary antibody (1:1000).^[22]^ Protein signals were detected with a chemiluminescent ECL^TM^ Detection Kit (WBKLS0100, Millipore), scanned using a Gel Doc system, and analyzed using Image Lab Software. Protein levels were quantified using Image J software.^[23]^ Rabbit anti-GAPDH antibodies were used as controls. Rabbit anti-MMP9, anti-TGFB1, and anti-CCND1 primary antibodies were used.

#### 2.5.4. Statistical analysis

Data from 3 independent biological experiments (each with 3 technical replicates) were analyzed using SPSS 20.0 and GraphPad Prism 9.0. Results are expressed as mean ± standard error of mean. After confirming normality (Shapiro–Wilk test) and homogeneity of variance (Levene test): One-way ANOVA with Tukey post hoc test (for multiple comparisons), Two-way ANOVA (for time/dose-dependent experiments), *P* < .05 was considered statistically significant (*P* < .05, *P < .01, **P < .001).

## 3. Results

### 3.1. Acquisition, screening and network construction of HL-RG bioactive compounds and related human targets

**Figure 2.**
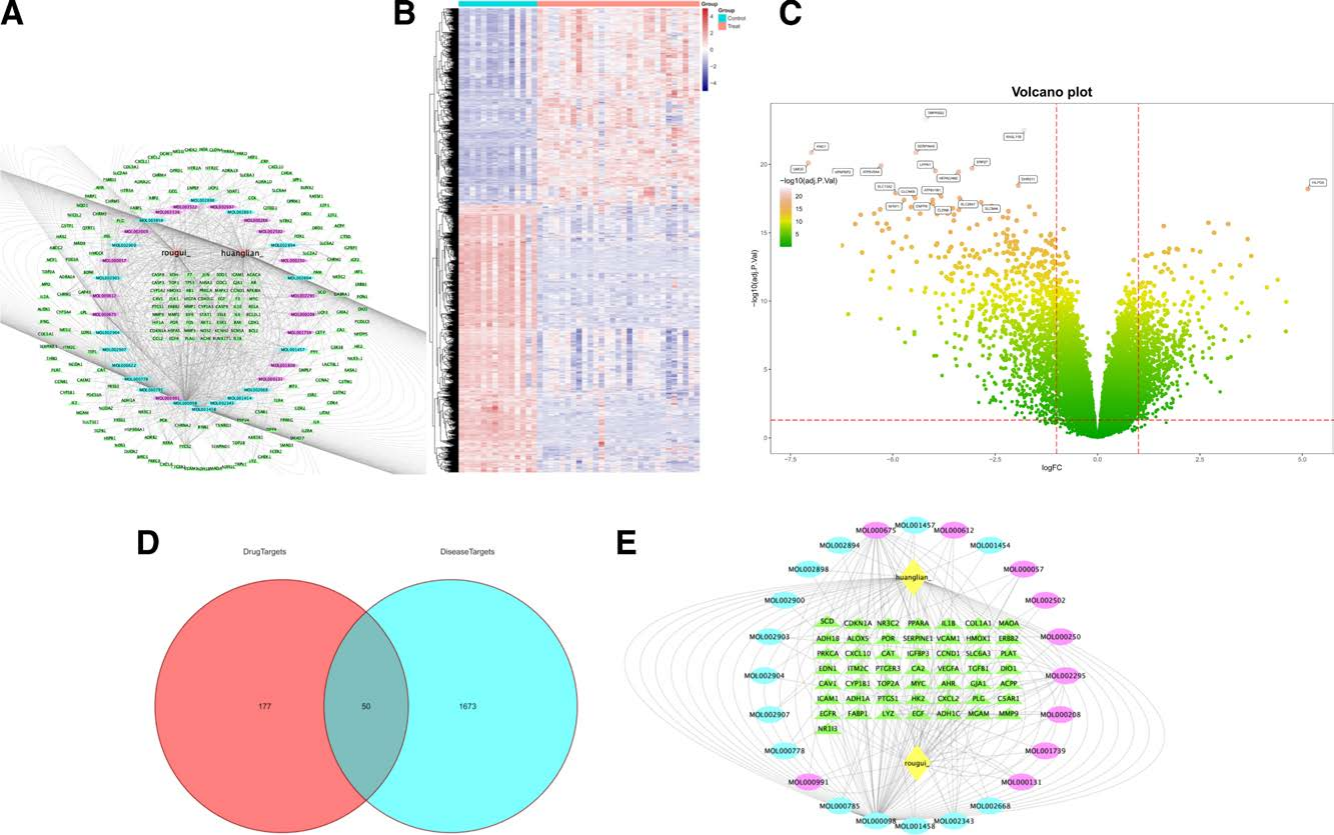
(A) TCM-active ingredients-targets network. (B) Gene heat map. (C) Volcano plot. (D) Venny plot. (E) TCM-active ingredients-specific targets network. TCM = traditional Chinese medicine.

Using “huanglian” and “rougui” as search terms in the TCMSP database, we identified 149 chemical constituents (49 from HL and 100 from RG). After screening, 25 bioactive components from HL (Table 1) and 17 from RG (Table 2) were selected.^[24]^ These components were associated with 185 human target proteins for HL and 76 for RG. After removing duplicates, 227 unique human targets were retained. The association network of TCM-bioactive components-target was constructed by using Cytoscape software (version 3.7.2) (Fig. 2A). The network diagram consists of 263 nodes and 480 edges. Comprises 2 TCM nodes, which are represented by red V-shaped nodes, There are 18 nodes of bioactive components in HL, which are represented by peach oval nodes; There are 16 nodes of bioactive components in RG, which are represented by blue oval nodes, There are 227 target nodes, which are represented by green triangular nodes. The correlation between TCM and bioactive components is represented by gray dotted lines, and 1 drug flavor corresponds to multiple components. The interaction between bioactive components and target protein is represented by gray dotted line, 1 component corresponds to multiple targets, and multiple components will act on the same target, which demonstrates the characteristics of multi-component and multi-target action of TCM.

**Table 1:**
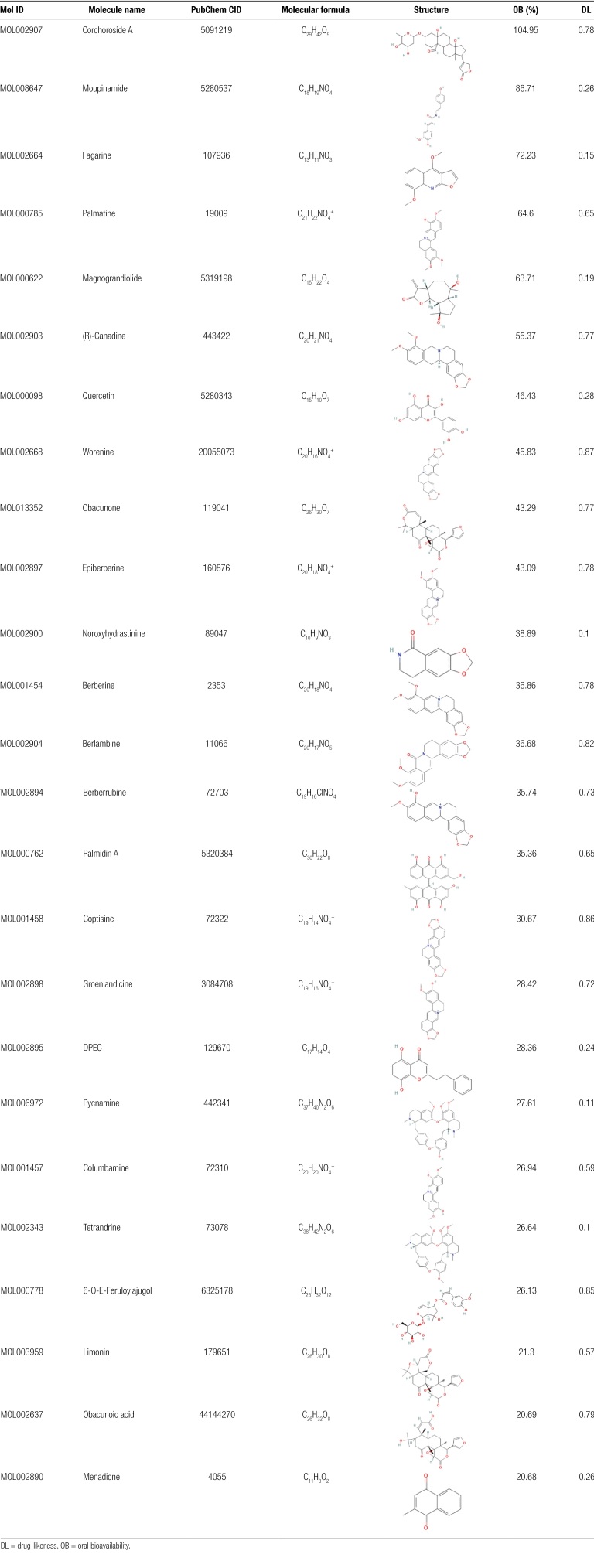
Bioactive components of *Coptidis Rhizoma*.

**Table 2:** Active ingredients of *Cinnamomi Cortex*.

Mol ID	Molecule name	PubChem CID	Molecular formula	OB (%)	DL
MOL000208	(+)-Aromadendrene	11095734	C_15_H_24_	55.74	0.1
MOL000612	(−)-α-Cedrene	6431015	C_15_H_24_	55.56	0.1
MOL003538	(+)-Ledene	10910653	C_15_H_24_	51.84	0.1
MOL000057	DIBP	6782	C_16_H_22_O_4_	49.63	0.13
MOL002697	Junipene	1796220	C_15_H_24_	44.07	0.11
MOL000131	Linoleic Acid	5280450	C_18_H_32_O_2_	41.9	0.14
MOL003522	(+)-Sativene	11275742	C_15_H_24_	37.41	0.1
MOL001739	Zoomaric acid	445638	C_16_H_30_O_2_	35.78	0.1
MOL000675	Oleic acid	445639	C_18_H_34_O_2_	33.13	0.14
MOL000266	β-Cubebene	93081	C_15_H_24_	32.81	0.11
MOL002003	(−)-Caryophyllene oxide	1742210	C_15_H_24_O	32.67	0.13
MOL001600	α-Ylangene	20055075	C_15_H_24_	29.47	0.12
MOL002502	(−)-Copaene	12303902	C_15_H_24_	24.08	0.12
MOL002225	Styrone	5315892	C_9_H_10_O	38.35	0.02
MOL000991	Cinnamaldehyde	637511	C_9_H_8_O	31.99	0.02
MOL000250	cis-Cinnamaldehyde	6428995	C_9_H_8_O	27.21	0.02
MOL002295	Cinnamic acid	444539	C_9_H_8_O_2_	19.68	0.03

DL = drug-likeness, OB = oral bioavailability.

### 3.2. Identification of RCC-specific related to HL-RG

We identified 1723 differentially expressed genes in RCC, including 996 downregulated and 727 upregulated genes. Gene expression patterns were visualized through: gene heat map (Fig. 2B) allows us to feel the expression patterns and laws of 1723 differential genes between RCC samples and normal samples more intuitively. Upregulated genes are represented in red, downregulated genes in blue, and the depth of color indicates the expression level. The figure shows the results of clustering the expression levels of 1723 genes (columns represent genes) and 43 samples including normal people and patients (rows represent samples). It is easy for us to observe the classification of gene groups and population samples in the diagram (tree structure above the boundary of the diagram and on the left of the boundary of the diagram). In the volcanic map of the gene (Fig. 2C), the horizontal axis is log fold change (*logFC*), showing the difference multiple, and the more the point deviates from the center, the greater the difference multiple; The vertical axis is *-log*_*10*_*(adj.P.Val*), which shows significance. Each point in the figure represents a detected gene, and the closer the point is to the top of the graph, the more significant the difference is. The differential genes in the disease samples are normally distributed, and the statistically significant Top 20 genes are highlighted on the map. These 20 genes contain 19 downregulated genes, TMPRSS2, RASL11B, SERPINA5, KNG1, UMOD, ATP6V0A4, ERP27, LPPR1, HEPACAM2, XPNPEP2, DHRS11, SLC13A2, ATP6V1B1, CLCNKB, SLC26A7, SFRP1, ENPP6, SLC9A4, and CLDN8. And 1 upregulated gene, HILPDA.

### 3.3. Identification of RCC-specific targets related to HL-RG and construction of the Chinese medicine-compound-target network

We identified 50 RCC-specific targets (Fig. 2D), comprising 28 upregulated (Table 3) and 22 downregulated genes (Table 4). The Chinese medicine-compound-target network was constructed using Cytoscape (v3.7.2) (Fig. 2E), with the following features: the network diagram consists of 76 nodes and 174 edges. Comprise 2 Chinese medicine nodes, which are represented by yellow diamond nodes; There are 14 nodes of bioactive components in HL, which are represented by blue oval nodes; There are 10 nodes of bioactive components in RG, which are represented by peach oval nodes; There are 50 specific target nodes, which are represented by green triangular nodes. The correlation between TCM and bioactive components is represented by gray dotted lines, and 1 drug flavor corresponds to multiple components. The interaction between bioactive components and specific target proteins of diseases is represented by gray dotted lines, and 1 component corresponds to multiple targets, and multiple components will act on the same target. The therapeutic effect of HL-RG on RCC was verified, and the possible effective components and targets were shown.

**Table 3:** Upregulated genes in specific targets.

Gene name	logFC	*P*-value	adj.*P*-value
IGFBP3	3.188126764	5.44E−19	2.26E−16
VEGFA	2.541368233	5.39E−14	4.33E−12
HK2	3.138818234	4.67E−13	2.77E−11
EGFR	1.589231162	8.05E−11	2.32E−09
CAV1	2.187554139	2.38E−10	5.86E−09
SCD	2.724795338	2.77E−09	4.97E−08
SLC6A3	3.517306376	4.97E−09	8.27E−08
TGFB1	1.330533188	1.45E−08	2.11E−07
AHR	1.112715896	3.23E−08	4.16E−07
CCND1	1.696288304	5.61E−08	6.68E−07
MYC	1.498752232	2.18E−07	2.14E−06
GJA1	1.465893063	6.33E−07	5.44E−06
CDKN1A	1.155261376	1.57E−06	1.19E−05
ALOX5	1.525346556	7.12E−06	4.47E−05
LYZ	1.618281354	7.73E−06	4.78E−05
ICAM1	1.26987265	8.09E−06	4.97E−05
TOP2A	1.371071992	9.40E−06	5.64E−05
PTGS1	1.612017102	1.29E−05	7.47E−05
C5AR1	1.203050829	2.81E−05	.000146732
EDN1	1.712819234	5.65E−05	.000266952
COL1A1	1.568330164	.000249084	.000964569
CXCL2	1.104492655	.000422688	.001539405
HMOX1	1.065536909	.000605065	.00209821
CXCL10	1.797396825	.000937587	.003073698
SERPINE1	1.641542489	.00287619	.008021441
IL1B	1.01740178	.004252183	.011256462
VCAM1	1.123204133	.008094309	.01954318
MMP9	1.090760596	.010485195	.024357455

logFC = log_2_ fold change.

**Table 4:** Downregulated genes in specific targets.

Gene name	logFC	*P*-value	adj.*P*-value
FABP1	−4.780858429	7.02E−17	1.48E−14
EGF	−4.418287045	9.99E−16	1.33E−13
ADH1C	−2.690497335	5.55E−15	6.08E−13
PLG	−5.539451327	2.88E−14	2.52E−12
ADH1B	−3.34642377	2.35E−12	1.11E−10
POR	−1.019297306	3.25E−12	1.45E−10
NR1I3	−1.13139671	2.69E−10	6.49E−09
ACPP	−2.878502795	6.57E−10	1.41E−08
CAT	−1.26201897	6.77E−10	1.45E−08
PPARα	−1.214144944	2.45E−09	4.48E−08
ADH1A	−1.115644513	1.85E−08	2.57E−07
ERBB2	−1.40201351	6.16E−08	7.25E−07
NR3C2	−1.297511223	6.30E−08	7.38E−07
PLAT	−1.759329821	1.62E−06	1.23E−05
PRKCA	−1.086495245	3.75E−06	2.55E−05
CA2	−1.1070129	3.78E−06	2.58E−05
PTGER3	−1.355821485	3.93E−05	.00019543
MGAM	−1.966215495	.001254383	.003940715
MAOA	−1.005403732	.008638908	.020658331
CYP1B1	−1.170348588	.018912469	.040347108
ITM2C	−1.170026632	.00011246	.000483879
DIO1	−5.307019362	2.55E−20	1.93E−17

logFC = log_2_ fold change.

### 3.4. Construction of PPI network and identification of hub genes

**Figure 3.**
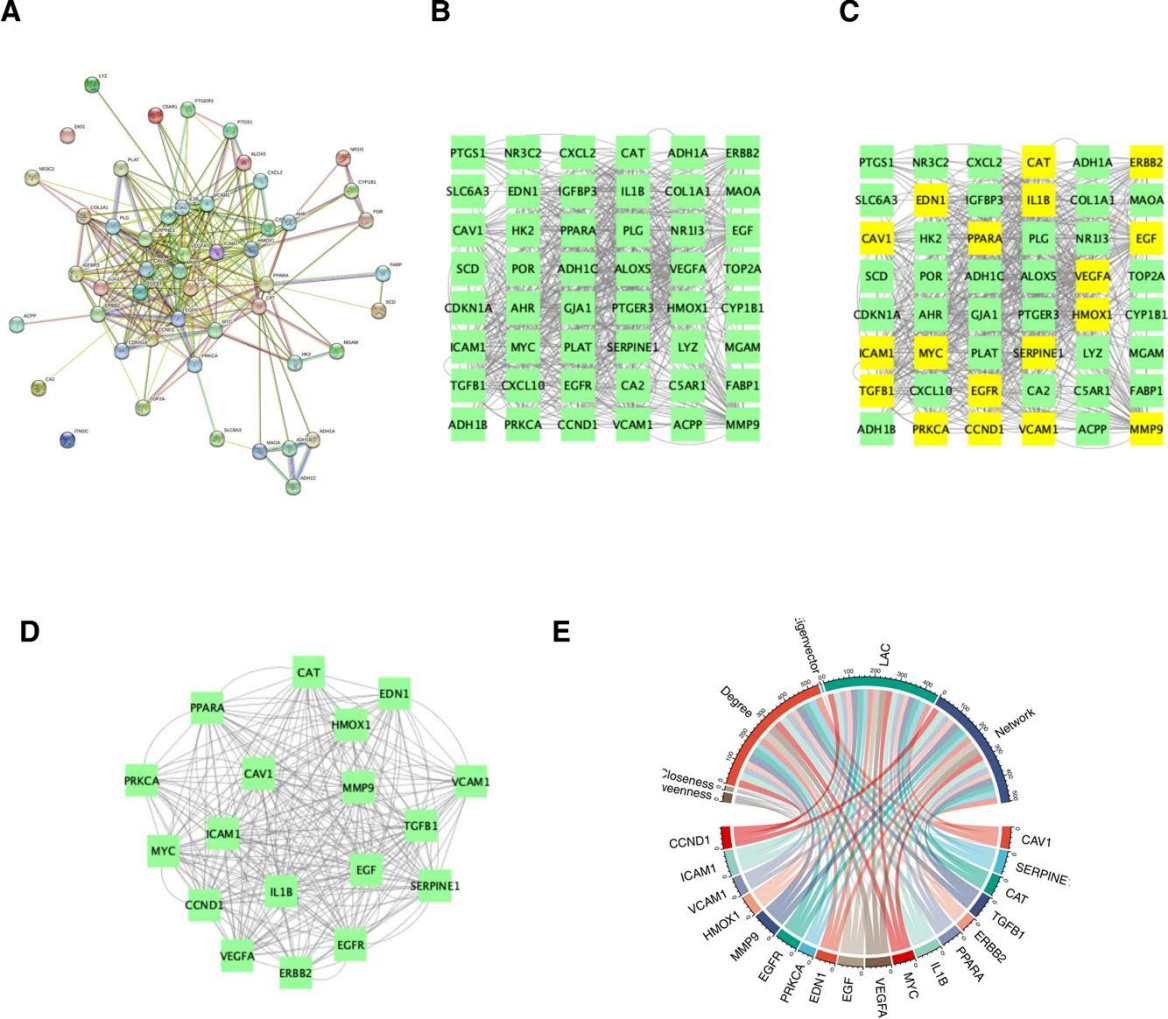
(A) The PPI network of 50 specific targets in the STRING database. (B) The PPI network of 50 specific targets in the Cytoscape software. (C) The PPI network of 50 specific targets in the Cytoscape software. (D) The PPI network of 18 specific targets in the Cytoscape software. (E) The chord diagram of 18 hub genes. PPI = protein–protein interaction.

The PPI network of these 50 candidate genes was generated (Fig. 3A) and visualized using Cytoscape (Fig. 3B). Through topological analysis in R Studio, we identified 18 hub genes (Fig. 3C and D). Their interactions were further illustrated through a chord diagram (R package, Fig. 3E). The network diagram (Fig. 3B) is an interactive diagram of the network relationship of 50 specific genes, which consists of 48 nodes and 1164 edges. The 48 nodes are specific targets (two genes are not involved in the interaction relationship and exist alone, and are not involved in the network diagram drawing), which are represented by green square nodes, and the mutual relationship between targets is represented by gray dashed lines. In the network diagram (Fig. 3D), the yellow square nodes represent the selected hub genes. In the network diagram (Fig. 3E), the network interaction of 18 key genes is shown, which consists of 18 nodes and 540 edges. The interaction and influence between targets show good interaction between targets, which indicates the possibility of multiple targets acting on the same pathway.

### 3.5. GO, KEGG analysis, and construction of related core target-action pathway network

**Figure 4.**
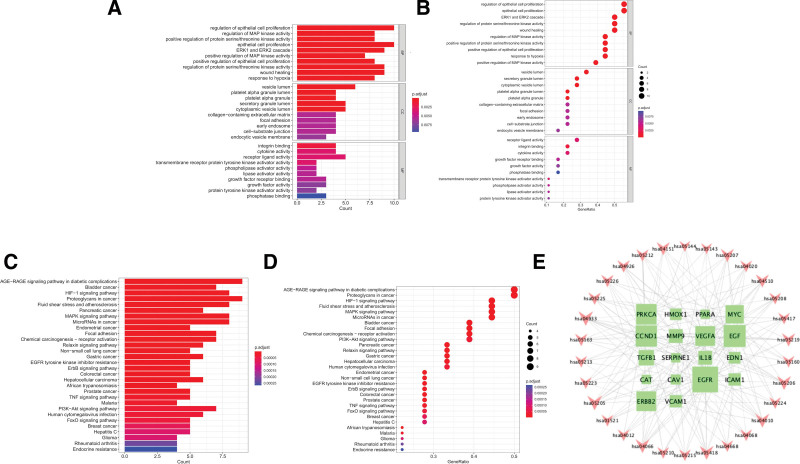
(A) The bar plot of GO analysis. (B) The bubble diagram of GO analysis. (C) The bar plot of KEGG analysis. (D) The bubble diagram of KEGG analysis. (E) Network visualization from bioinformatics analysis highlighting the detailed interactions of hub genes-KEGG. GO = gene ontology, KEGG = Kyoto Gene and Genome Encyclopedia.

We obtained a total of 1275 GO terms of the BP, including 1216 items of BP, primarily involving regulation of epithelial cell proliferation, regulation of MAP kinase activity, positive regulation of protein serine/threonine kinase activity, etc. There are 35 items of CC, the majority of which include vesicle lumen, platelet alpha granule lumen, platelet alpha granule, etc. There are 24 items of molecular function, the majority of which involve integrin binding, cytokine activity, receptor-ligand activity, etc (Fig. 4A and B).^[25]^ The results of enrichment for the TOP 30 pathways were displayed as a bubble diagram (Fig. 4C and D). KEGG pathways associated with RCC include AGE-RAGE signaling pathway in diabetic complications, Proteoglycans in cancer, HIF-1 signaling pathway, etc. Among them, AGE-RAGE signaling pathway in diabetic complications involves 9 hub targets, including 7 upregulated genes (VEGFA, IL1B, ICAM1, VCAM1, TGFB1, SERPINE1, CCND1, EDN1) and 1 downregulated gene (PRKCA). Proteoglycans in cancer signaling pathway involves 9 hub targets, all of which are upregulated genes (ERBB2, EGFR, VEGFA, MYC, CAV1, MMP9, TGFB1, CCND1, PRKCA). HIF-1 signaling pathway involves 8 hub targets, including 5 upregulated genes (HMOX1, EGFR, VEGFA, SERPINE1, EDN1) and 3 downregulated genes (ERBB2, EGF, PRKCA). The interaction network of hub genes – KEGG pathway is constructed (Fig. 4E), which consists of 48 nodes and 181 edges. Including 18 core targets, represented by green square nodes, the size of which shows the number of related pathways; There are 30 nodes in KEGG pathway, which are represented by red V-shaped nodes. The correlation between target and KEGG pathway is indicated by gray dotted line. This multi-target/multi-pathway pattern suggests HL-RG’s therapeutic potential in RCC through modulation of inflammation, immune response, cell cycle, and lipid metabolism.^[26]^

### 3.6. Molecular docking validation

**Figure 5.**
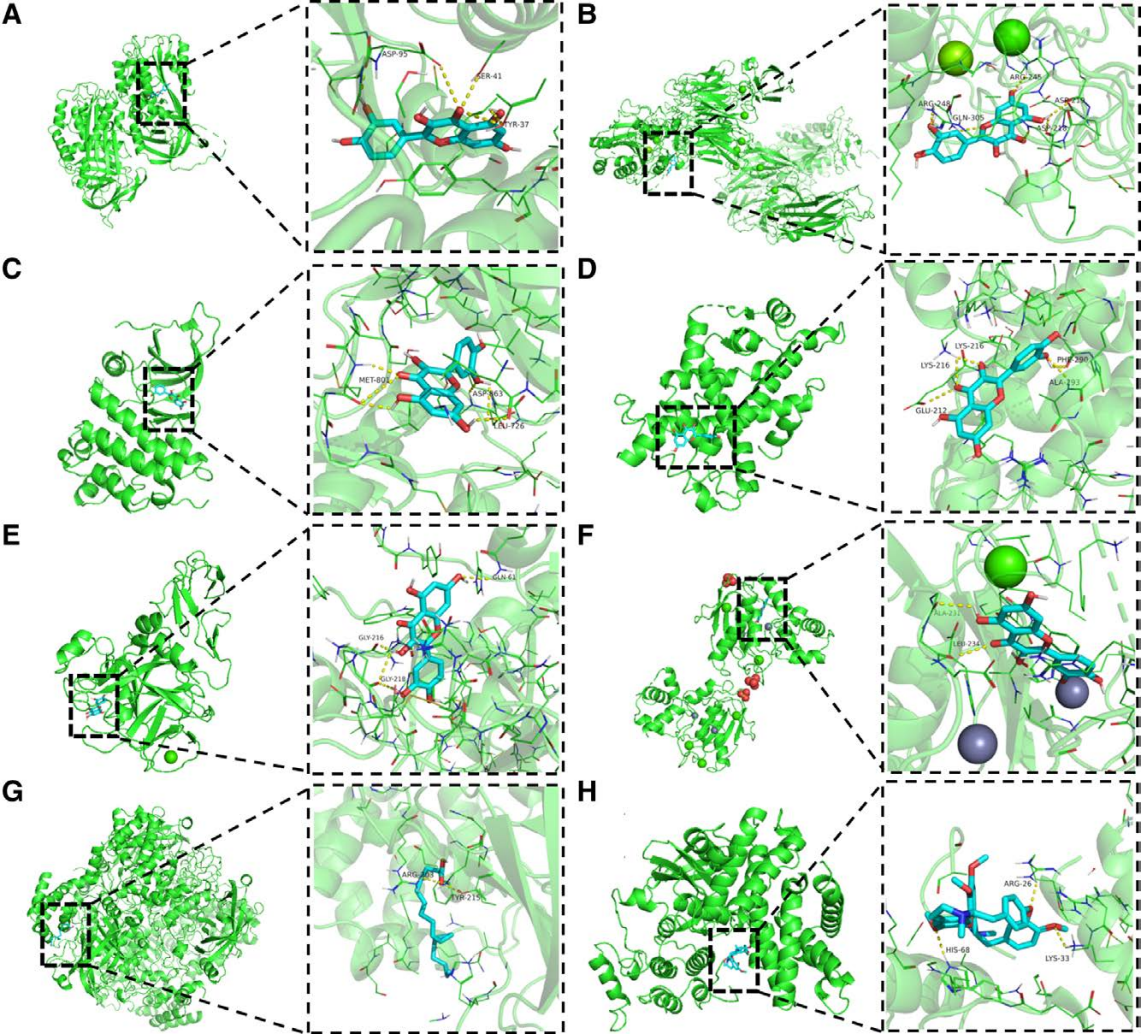
Molecular docking diagram of chemical composition to target. (A) SERPINE1-quercetin; (B) TGFB1-quercetin; (C) ERBB2-quercetin; (D) PPARα-quercetin; (E) EGF-quercetin; (F) MMP9-quercetin; (G) CAT-oleic acid; (H) CCND1-tetrandrine.

We performed molecular docking using 18 hub targets as receptors and their corresponding active components as ligands. Binding stability was evaluated based on binding energy. Key interactions were visualized with PyMOL (Fig. 5), including: QUE (MOL000098) with SERPINE1, TGFB1, ERBB2, PPARα, EGF, and MMP9 (Matrix metalloproteinase-9), OA (MOL000675) with CAT, TET (MOL002343) with CCND1. Docking results (Table 5) confirmed strong binding affinity between these bioactive compounds and their targets (binding energy < -7 kcal/mol).^[27]^ This validates QUE, OA, and TET as the primary anti-tumor components of HL-RG,^[28,29]^ demonstrating their potential therapeutic effects against RCC.

**Table 5:** Docking results of hub genes and active compounds.

Mol Id	Molecule name	Hub genes	PDB ID	Affinity (kcal/mol)	Hydrogen bond	Amino acid residues
MOL000098	Quercetin	CAV1	7SC0	−6.6	2	HIS-153; THR-154
		SERPINE1	7AQF	−8.7	5	ASP-95(2); SER-41; TYR-37(2)
		TGFB1	6OM2	−8.5	5	ARG-248; GLN-305; ARG-245; ASP-219; ASP-218
		ERBB2	7PCD	−8.4	6	MET-801(3); ASP-863; LEU-726(2)
		PPARα	6KAX	−8.0	5	LYS-216; LYS-216; GLU-212; PHE-290; ALA-293
		IL1B	5R8Q	−7.1	6	PRO-87; LYS-65; TYR-68; LYS-63; ASN-7(2)
		MYC	4Y7R	−8.1	7	FHE-137(2); TRP-95; ASN-136; LYS-221; CYS-309
		VEGFA	4KZN	−8.0	4	ASN-62; CYS-68; GLY-59; LYS-48
		EGF	1XKA	−9.3	4	GLY-216; GLY-218(2); GLN-61
		PRKCA	4DNL	−8.0	7	THR-228(2); LYS-230; ASP-235(3);
		EGFR	5UGA	−8.1	4	MET-793(2); LEU-718; ASN-842
		MMP9	6ESM	−9.0	2	ALA-231; LEU-234
		HMOX1	1N45	−8.2	3	GLN-38; HIS-25; THR-135
		VCAM1	1VCA	−6.9	1	LYS-147
		ICAM1	3BQN	−8.4	1	LE-258
		CCND1	2W96	−9.4	6	ASP-99(2); LYS-35; ASN-145; ALA-16; LYS-142
MOL000675	Oleic acid	SERPINE1	7AQF	−4.3	1	ASN-31
		CAT	1DGF	−5.6	2	ARG-203; TYR-215
		ERBB2	7PCD	−5.6	2	ILE-767
		PPARα	6KAX	−4.7	3	RG-209; ARG-209; GLY-296
		EDN1	1T7H	−3	2	SER-2
MOL002343	Tetrandrine	TGFB1	6OM2	−8.3	2	ALA-282, LYS-410
		CCND1	2W96	−8.5	3	HIS-68; ARG-26; LYS-33

PDB = Protein Data Bank.

### 3.7. Validation of hub target gene expression

**Figure 6.**
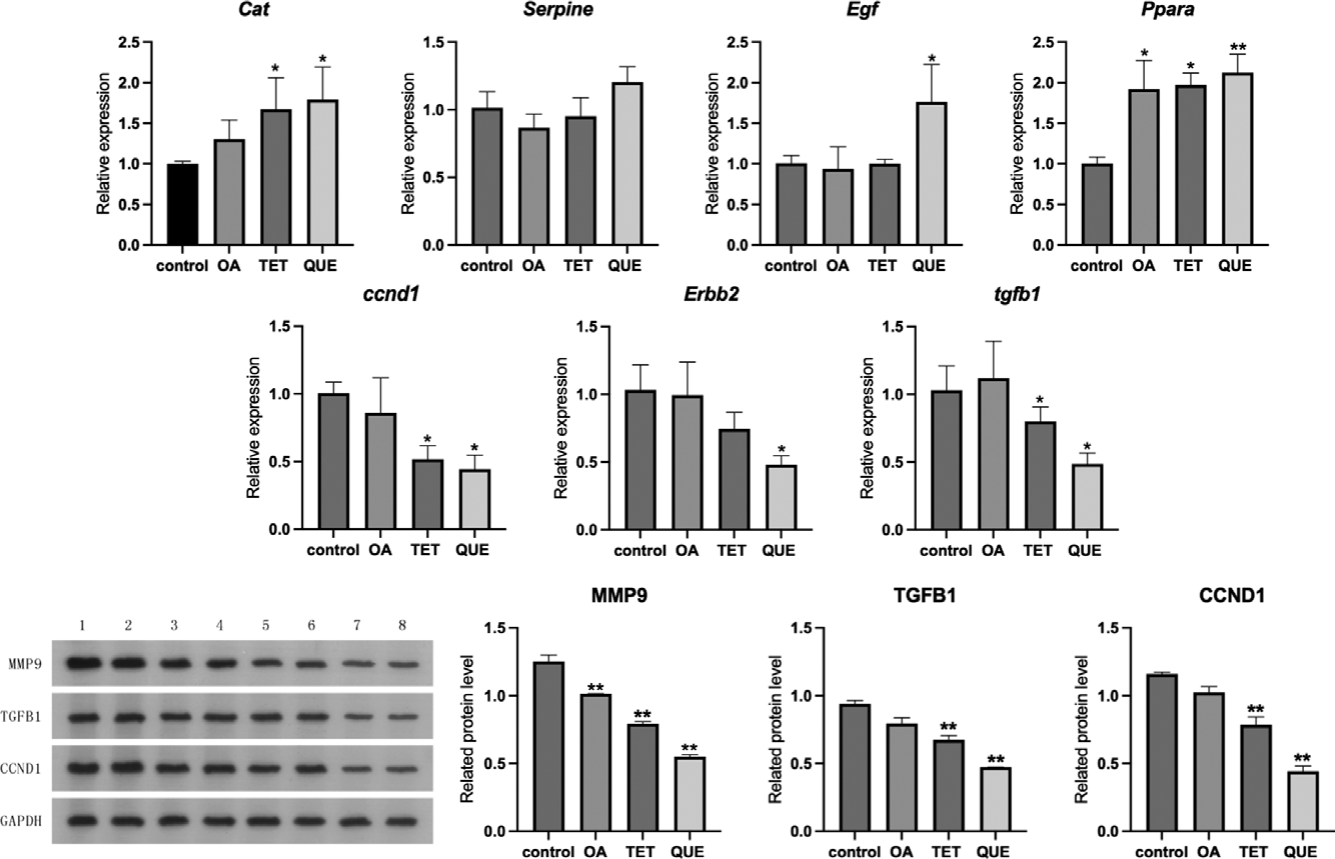
(A) qRT-PCR of related genes mRNA levels in different groups. (B) Western blot of related protein levels in different groups. *P ≤ .05, **P ≤ .01, compared to control group. qRT-PCR = quantitative real-time polymerase chain reaction.

The mRNA expression of 7 hub genes (*Tgf-β1, Ccnd1, Cat, Erbb2, Ppar*α*, Egf, serpine 1*) were quantified by qRT-PCR. Compared with the control group, the relative expression of Cat mRNA in TET group and QUE group increased significantly (*P ≤ .05*), and the relative expression of *Egf* mRNA in QUE group increased significantly (*P ≤ .05*). The relative expression of *Ccdn1* and *Tgf-b1* mRNA in TET group and QUE group decreased significantly (*P ≤ .05*), and the relative expression of *Erbb2* mRNA in QUE group decreased significantly (*P ≤ .05*), as shown in Figure 6A.

The Western blot results showed that compared with the control group, the levels of MMP9-related protein in OA group, TET group and QUE group were significantly decreased (*P ≤ .01*), the levels of TGFB1-related protein in TET group and QUE group were significantly decreased (*P ≤ .01*), and the levels of CCND1-related protein in TET group and QUE group were significantly decreased (*P ≤ .01*), as shown in Figure 6B (technical duplicates shown for each biological sample). The experimental results are consistent with those of qRT-PCR.

## 4. Discussion

QUE interacts with 16 targets, whereas OA and TET bind to 5 and 2 targets, respectively. Molecular docking confirmed their strong binding affinity to hub genes (binding energy < -7 kcal/mol). QUE is a flavonol compound that possesses diverse biological activities. Numerous studies have demonstrated that QUE significantly inhibits the progression of lung cancer, liver cancer, cervical cancer, breast cancer, and other malignancies by inducing tumor cell apoptosis and inhibiting tumor cell proliferation, migration, and invasion. In addition, it has been discovered that QUE can collaborate with certain chemotherapy medications to play a more effective role and reduce multidrug resistance, thereby improving patients’ quality of life. QUE exerts anti-tumor effects through multiple mechanisms and has broad clinical anti-tumor treatment application potential.^[30–33]^ OA is exhibits low toxicity and can combat SFA-induced toxicity through alleviation of cell apoptosis, endoplasmic reticulum stress (ER stress) and lipids metabolism disorder, and it is able to effectively improve autophagy dysfunction under the context of both PA and ER stress inducer induced lipotoxicity, and OA-mediated regulation of lysosomal dysfunction via TFEB is critical, suggesting that the regulation of ER stress-autophagy axis is a critical mechanism in OA driven protection in NAFLD.^[34]^ TET is a natural bisbenzylisoquinoline alkaloid isolated from *Stephaniae tetrandrae*, which has anti-inflammatory and anti-tumor activities. By inhibiting STING/TBK1/NF-κB signaling pathway, the inflammation of macrophages attacked by oxLDL can be reduced, and the atherosclerosis of ApoE mice fed with HFD can be alleviated.^[35]^ It can selectively inhibit the expression of COX-2 gene and reduce the level of cytokines in mice, thus reducing inflammation and improving osteoarthritis.^[36]^ In vitro, TET strongly inhibits cell proliferation; in vivo, it suppresses leukemia progression.^[37]^ It can inhibit the expression of TNF-α in cancer tissues and blood, reduce the release of inflammatory factors and chemokines, and reduce the proliferation of cancer cells.^[38]^

CAT is an antioxidant enzyme that can decompose hydrogen peroxide and protect cells from oxidative damage. In the occurrence of cancer, CAT is related to the cellular oxidative stress response, can inhibit the activation of NF-κB, and promote cell apoptosis. PPARα is a nuclear hormone receptor with high expression levels in various organs such as the liver, kidney, and heart, especially in organs with active gluconeogenesis and catabolism. Due to the strong proliferation ability of cancer cells, the neovascularization of tumors cannot meet the growth needs of tumor cells, resulting in a large amount of necrosis and a decrease in PPARα expression levels.^[39]^ PPARα agonists can increase the expression level of PPARα in the liver, reduce oxidative stress and inflammatory responses, and alleviate organ damage.^[40,41]^ In our study, QUE group, TET group, and OA group can all upregulate the expression of CAT and PPARα genes, among which the upregulating effects of QUE group and TET group are significant. NF-κB serves as a central hub integrating multiple signaling pathways, playing a regulatory and promoting role in the expression of angiogenesis-related factors, including MMP9. The overexpression of MMP9 plays a critical role in the invasion and metastasis of tumor cells. MMP9 is a member of the matrix metalloproteinase family, and under normal physiological conditions, the expression level of MMP9 in cells is low. However, when cells undergo inflammatory reactions or tumor growth, its expression level significantly increases. Elevated MMP9 levels promote tumor invasion, establishing a feed-forward loop that exacerbates tumor development. Therefore, reducing the expression level of MMP9 can inhibit the invasion and proliferation of tumor cells.^[42,43]^ PPARα can inhibit the expression of NF-κB, which in turn can regulate its downstream protein MMP9, suppressing the expression of MMP9.

The results of this study show that the levels of MMP9 in the QUE group, TET group, and OA group were significantly reduced. The above research results suggest that QUE, TET, and OA may play a therapeutic role in RCC by regulating the NF-κB signaling pathway.

Beyond the NF-κB pathway, ERBB2 (HER2) overexpression is implicated in tumorigenesis. ERBB2 gene is abnormally highly expressed in various tumor tissues, including breast cancer, ovarian cancer, pancreatic cancer, and others. Targeted inhibition of ERBB2 expression can suppress the growth and invasion of breast cancer cells. Multiple factors can induce apoptosis in gastric cancer cells by downregulating ERBB2 expression, inhibiting cell proliferation and growth. Numerous studies show that ERBB2 can regulate the proliferation and invasion of cancer cells by modulating the PI3K/AKT pathway. The PI3K/AKT/HIF-1α signaling pathway is activated under hypoxic conditions, where phosphatidylinositol 3-kinase (PI3K) is stimulated and binds with its downstream serine/threonine kinase (AKT), leading to AKT phosphorylation and increased HIF-1α activity. This initiates the transcription of downstream target genes, resulting in increased cell proliferation and decreased apoptosis. This pathway is related to the level of cellular glycolysis, with hexokinase II (HKII)/glucose transporter 1 (GLUT1) and lactate dehydrogenase (LDHA) potentially being downstream effectors of this pathway. Under hypoxic conditions, epidermal growth factor (EGF) activates the PI3K/AKT pathway and participates in the regulation of glycolysis through HIF-1α; inhibiting the PI3K/AKT/HIF-1α pathway can significantly reduce cellular glycolysis. The PI3K signaling pathway plays a role in cell proliferation and apoptosis. The PI3K/AKT/HIF-1α signaling pathway is closely related to the occurrence and development of tumors, and inhibiting this pathway can suppress cancer cell proliferation and promote apoptosis.^[44]^ In our study, 3 active components all had varying degrees of downregulating effects on ERBB2 expression, with the QUE group showing significant downregulation. This suggests that QUE may have therapeutic effects on RCC by modulating the PI3K/AKT signaling pathway.

CCND1 gene expresses the CCND1 protein, which promotes the cell cycle progression from the G1 phase to the S phase. After the formation of the IgH/CCND1 fusion gene, overexpression of CCND1 leads to cell cycle disorders and uncontrolled cell growth. Studies have shown that CCND1 overexpression is found in various types of tumors, including not only lymphomas but also breast cancer, bladder cancer, parathyroid tumors, lymphomas, melanomas, lung cancer, and centrocytic lymphomas. CCND1 itself has the function of repairing damaged DNA, which triggers resistance to chemotherapy drugs and helps cancer cells to rapidly repair damage. Overexpression of CCND1 can also secrete a vascular endothelial growth factor, providing nutrients for metastasized cancer cells. Research results indicate that both the QUE group and the TET group have a significant downregulation trend in the CCND1 gene, which can inhibit the growth of RCC.^[45]^

TGFB1 gene acts as a tumor suppressor by activating cell apoptosis and reducing the expression of genes encoding vascular endothelial growth factor. Overexpression of this gene decreases the immune response to cancer cells, thereby promoting the carcinogenic process. Additionally, the TGFB1 gene promotes epithelial-mesenchymal transition, facilitating the metastasis of cancer cells. The Transforming Growth Factor β signaling pathway (TGF-β signaling pathway) plays a crucial role in cellular homeostasis, participating in numerous BPs such as controlling cell growth, differentiation, or migration, angiogenesis, and even regulating apoptosis. Disruptions in gene expression within the TGF-β pathway are one of the factors involved in the development of many cancers, making the study of this pathway very important for cancer research. In our study, both the QUE and TET groups showed a significant downregulation trend in the TGFB1 gene, suggesting that the therapeutic effects of QUE and TET on RCC may be related to the TGF-β signaling pathway.^[46]^

Collectively, our data suggest that HL-RG may treat RCC by modulating TGFB1/CCND1/MMP9/ERBB2/CAT/PPARα networks via NF-κB, PI3K/AKT, and TGF-β pathways. In this study, the potential target of HL-RG in the treatment of gastric cancer was studied Through PPI network analysis and molecular pairing, TGFB1, CCND1, MMP9, ERBB2, CAT, and PPARα may be the core targets of HL-RG in the treatment of RCC. At the same time, through in vitro experiments, it was found that its active components OA, TET and QUE can down-regulate the genes TGFB1, CCND1, ERBB2, and MMP9 that are highly expressed in RCC, and upregulate the genes CAT and PPARα that are poorly expressed in RCC. The related pathways may include the NF-κB signaling pathway, the PI3K/AKT/HIF-1α signaling pathway, and the TGF-β signaling pathway, among others, which require further experimental research to confirm.

## 5. Conclusion

TCM has the characteristics of natural source, multi-component, multi-pathway, and multi-target, and has advantages in the treatment of complex diseases. This study systematically elucidates, for the first time, the mechanism by which HL-RG treats RCC, and OA, TET and QUE were identified as the main active components of HL-RG in the treatment of RCC. The mechanism of HL-RG in the treatment of RCC involves multiple targets. Molecular docking demonstrated strong binding affinities of OA, TET, and QUE to core targets. qRT-PCR and Western blot analyses confirmed that the expression levels of TGFB1, CCND1, MMP9, ERBB2, PPARα and CAT aligned with bioinformatics predictions. Advances in molecular technologies have identified numerous potential RCC biomarkers, offering opportunities to refine prognostic assessments. In our work, genes with downregulated expression were hypothesized as tumor suppressors, while upregulated genes were potentially oncogenic in RCC. This is of great clinical significance for early diagnosis, treatment, and prognosis evaluation of RCC. Network pharmacology provides a novel and systematic analysis method for the study of Chinese herbal medicine.

Although network pharmacology has provided a novel direction for the clinical application of TCM, certain limitations remain. Our study has several shortcomings that warrant further investigation. Subsequent research should employ mass spectrometry, liquid chromatography, and animal experiments to: Identify and quantify the bioactive components of HL-RG, validate the predicted targets and pathways, and elucidate the precise mechanisms underlying HL-RG’s therapeutic effects against RCC. These efforts will strengthen the scientific foundation for clinical translation.

## Author contributions

**Conceptualization:** Mingjun Chen, Liyuan Tan.

**Data curation:** Liyuan Tan, Lingling Song.

**Formal analysis:** Liyuan Tan.

**Funding acquisition:** Dongmei Duan.

**Investigation:** Mingjun Chen, Dongmei Duan.

**Methodology:** Jin Wang, Liyuan Tan.

**Project administration:** Mingjun Chen, Jin Wang.

**Resources:** Dongmei Duan, Liyuan Tan.

**Software:** Jin Wang, Liyuan Tan.

**Supervision:** Dongmei Duan.

**Validation:** Mingjun Chen, Dongmei Duan.

**Visualization:** Jin Wang.

**Writing – original draft:** Liyuan Tan, Lingling Song.

**Writing – review & editing:** Liyuan Tan.
